# Supplementary material for: Nphos: Database and Predictor of Protein N-phosphorylation
Source: Genomics Proteomics Bioinformatics. 2024 Apr 10;22(3):qzae032. doi: 10.1093/gpbjnl/qzae032 (PMC12016571; doi:10.1093/gpbjnl/qzae032)
Supplement: qzae032_Supplementary_Data [file qzae032_supplementary_data.zip › Table S3_final version20240326.docx]

**Table S3 The performance of GBDT models before and after the hyperparameter fine-tuning**

| **Performance (%)** | **His** | | **Lys** | | **Arg** | |
| --- | --- | --- | --- | --- | --- | --- |
|  | **Before** | **After** | **Before** | **After** | **Before** | **After** |
| Accuracy | 88.55 ± 0.98 | 88.84 ± 1.05 | 89.45 ± 0.64 | 89.31 ± 0.72 | 88.82 ± 0.96 | 88.98 ± 0.92 |
| AUC | 90.00 ± 1.50 | 90.65 ± 1.43 | 91.27 ± 0.88 | 91.54 ± 0.95 | 91.33 ± 1.30 | 91.78 ± 1.10 |
| F1-score | 81.82 ± 1.77 | 82.21 ± 1.83 | 83.91 ± 1.06 | 83.42 ± 1.24 | 84.27 ± 1.47 | 84.14 ± 1.47 |
| MCC | 65.10 ± 3.25 | 65.98 ± 3.42 | 69.17 ± 1.99 | 68.56 ± 2.27 | 69.57 ± 2.75 | 69.77 ± 2.70 |
| Precision | 86.66 ± 1.54 | 87.32 ± 1.77 | 88.52 ± 1.11 | 88.86 ± 1.10 | 87.86 ± 1.39 | 88.79 ± 1.31 |
| Sensitivity or recall | 61.33 ± 3.86 | 61.56 ± 3.80 | 65.14 ± 2.47 | 63.13 ± 2.75 | 67.76 ± 3.33 | 65.75 ± 3.24 |
| Specificity | 96.52 ± 0.66 | 96.81 ± 0.76 | 96.98 ± 0.58 | 97.38 ± 0.48 | 96.19 ± 0.81 | 97.02 ± 0.67 |

*Note*: Before represents the model performance before optimizing parameters; After represents the model performance after optimizing parameters.
