# Supplementary material for: Nphos: Database and Predictor of Protein N-phosphorylation
Source: Genomics Proteomics Bioinformatics. 2024 Apr 10;22(3):qzae032. doi: 10.1093/gpbjnl/qzae032 (PMC12016571; doi:10.1093/gpbjnl/qzae032)
Supplement: qzae032_Supplementary_Data [file qzae032_supplementary_data.zip › Table S4_final version20240326.docx]

**Table S4 The MS raw data of protein *N*-phosphorylation**

| **PhosphoAA** | **Years** | **TaxID** | **Species** | **Instrument** | **PXID** | **Data size (Gb)** | **Ref. (PMID)** |
| --- | --- | --- | --- | --- | --- | --- | --- |
| pArg | 2012 | 224308 | *B. subtilis* (strain 168) | LTQ Orbitrap Velos | -- | -- | [22517742](https://www.ncbi.nlm.nih.gov/pubmed/22517742) |
| pArg | 2013 | 224308 | *B. subtilis* (strain 168) | LTQ Orbitrap Velos; Exactive | PXD000273 | 21.7 | [24263382](https://www.ncbi.nlm.nih.gov/pubmed/24263382) |
| pArg | 2014 | 224308 | *B. subtilis* (strain 168) | LTQ Orbitrap Velos | PXD000560 | 7.65 | [24825175](https://www.ncbi.nlm.nih.gov/pubmed/24825175) |
| pArg | 2016 | 224308 | *B. subtilis* (strain 168) | LTQ Orbitrap Velos | PXD003305 | 21.5 | [27749819](https://www.ncbi.nlm.nih.gov/pubmed/27749819) |
| pArg | 2017 | 93062 | *S. aureus* (strain COL) | LTQ Orbitrap Velos; LTQ Orbitrap Elite | PXD007167 | 474 | [29183913](https://pubmed.ncbi.nlm.nih.gov/29183913/) |
| pHis, pLys, pArg | 2017 | 9606 | HeLa cells, human | Orbitrap Fusion | -- | -- | [202820v1](https://www.biorxiv.org/content/10.1101/202820v1) |
| pArg | 2018 | 93062 | *S. aureus* (strain COL) | LTQ Orbitrap Velos; LTQ Orbitrap Elite | PXD009874 | 48 | [30358407](https://www.ncbi.nlm.nih.gov/pubmed/30358407) |
| pHis | 2018 | 83333 | *E. coli strain* K12 | Q Exactive | PXD008369 | 16.5 | [29377012](https://www.ncbi.nlm.nih.gov/pubmed/29377012) |
| pHis | 2019 | 7955 | *Danio rerio* | LTQ Orbitrap Velos | PXD012735 | 7.38 | [30864180](https://www.ncbi.nlm.nih.gov/pubmed/30864180) |
| pLys | 2019 | 83333 | *E. coli strain* K12 | Q Exactive | PXD012682 | 16.6 | [30993368](https://www.ncbi.nlm.nih.gov/pubmed/30993368) |
| pHis, pLys, pArg | 2019 | 9606 | HeLa cells, human | Orbitrap Fusion | PXD012188 | 103 | [31433507](https://www.ncbi.nlm.nih.gov/pubmed/31433507) |
| pHis, pLys, pArg | 2019 | 9606 | HeLa cells, human | LTQ Orbitrap Velos; Exactive | -- | -- | [691352](https://doi.org/10.1101/691352) |
| pHis | 2020 | 9606 | HeLa cells, human | Orbitrap Fusion Lumos | -- | -- | [32966065](https://www.ncbi.nlm.nih.gov/pubmed/32966065) |
| pHis, pLys, pArg | 2020 | 9606 | Jurkat cells, human | Orbitrap Fusion Lumos | PXD009696 | 30 | [s11426](https://link.springer.com/article/10.1007/s11426-019-9656-7) |
| pHis, pLys, pArg | 2020 | 83333 | *E. coli strain* K12 | Orbitrap Fusion Lumos | PXD017423 | 10.9 | [33277485](https://www.ncbi.nlm.nih.gov/pubmed/33277485) |
| pHis, pLys, pArg | 2020 | 9606 | HeLa cells, human | Orbitrap Fusion Lumos | PXD021067 | 38.6 | [33277485](https://www.ncbi.nlm.nih.gov/pubmed/33277485) |
| pArg | 2022 | 246196 | *M. smegmatis* | Q Exactive | PXD025324 | -- | [36214676](https://pubmed.ncbi.nlm.nih.gov/36214676) |

*Note*: AA, amino acid; pHis, pLys, and pArg indicate phosphorylation of histidine, lysine, and arginine, respectively. *B*. *subtilis*, *Bacillus subtilis*; *S*. *aureus*, *Staphylococcus aureus*; *M*. *smegmatis*, *Mycolicibacterium smegmatis*; LTQ, linear ion trap quadrupole; Q, quadrupole.
