# Supplementary material for: Nphos: Database and Predictor of Protein N-phosphorylation
Source: Genomics Proteomics Bioinformatics. 2024 Apr 10;22(3):qzae032. doi: 10.1093/gpbjnl/qzae032 (PMC12016571; doi:10.1093/gpbjnl/qzae032)
Supplement: qzae032_Supplementary_Data [file qzae032_supplementary_data.zip › Supplementary material captions.docx]

**Supplementary material**

**Figure S1 Protein length distribution of *N*-/*O*-phosphorylation in the proteomes of eukaryotes and prokaryotes**

The dotted line represents the mean of the overall distribution of protein length.

**Figure S2 Correlation analyses between amino acid usage and the occurrence rate of phosphorylation**

**A.** Results for eukaryotes. **B.** Results for prokaryotes.

**Figure S3 Sequential features around the pHis/pLys/pArg sites**

Logs are calculated in base 10. The conserved motifs were detected using rmotifx [score = ∑−log(*P*); score ≥ 5, occurrence ≥ 10].

**Table S1 Classifiers optimal results**

**Table S2 The fine-tuning of hyperparameters**

**Table S3 The performance of GBDT models before and after the hyperparameter fine-tuning**

**Table S4 The MS raw data of protein *N*-phosphorylation**

**Table S5 Optimal parameters of the classifiers**
