# Supplementary material for: Nphos: Database and Predictor of Protein N-phosphorylation
Source: Genomics Proteomics Bioinformatics. 2024 Apr 10;22(3):qzae032. doi: 10.1093/gpbjnl/qzae032 (PMC12016571; doi:10.1093/gpbjnl/qzae032)
Supplement: qzae032_Supplementary_Data [file qzae032_supplementary_data.zip › Table S2_final version20240326.docx]

**Table S2 The fine-tuning of hyperparameters**

| **Hyperparameters** | **Value list** | **Optimal parameter** | | |
| --- | --- | --- | --- | --- |
|  |  | **pHis** | **pLys** | **pArg** |
| learning_rate | [0.1, 0.3, 0.5, 0.6, 0.7] | 0.1 | 0.1 | 0.1 |
| subsample | [0.9, 1] | 0.9 | 1 | 0.9 |
| n_estimator | [170, 180, 190] | 170 | 170 | 170 |
| max_depth | [11, 12, 13, 16, 17, 18] | 11 | 11 | 11 |
| min_samples_leaf | [1, 2, 75, 76, 77] | 2 | 1 | 1 |
| min_samples_split | [300, 400, 402, 500] | 500 | 300 | 300 |

*Note*: pHis, pLys, and pArg indicate phosphorylation of histidine, lysine, and arginine, respectively.
