# Supplementary figures and images for: Nphos: Database and Predictor of Protein N-phosphorylation
Source: Genomics Proteomics Bioinformatics. 2024 Apr 10;22(3):qzae032. doi: 10.1093/gpbjnl/qzae032 (PMC12016571; doi:10.1093/gpbjnl/qzae032)

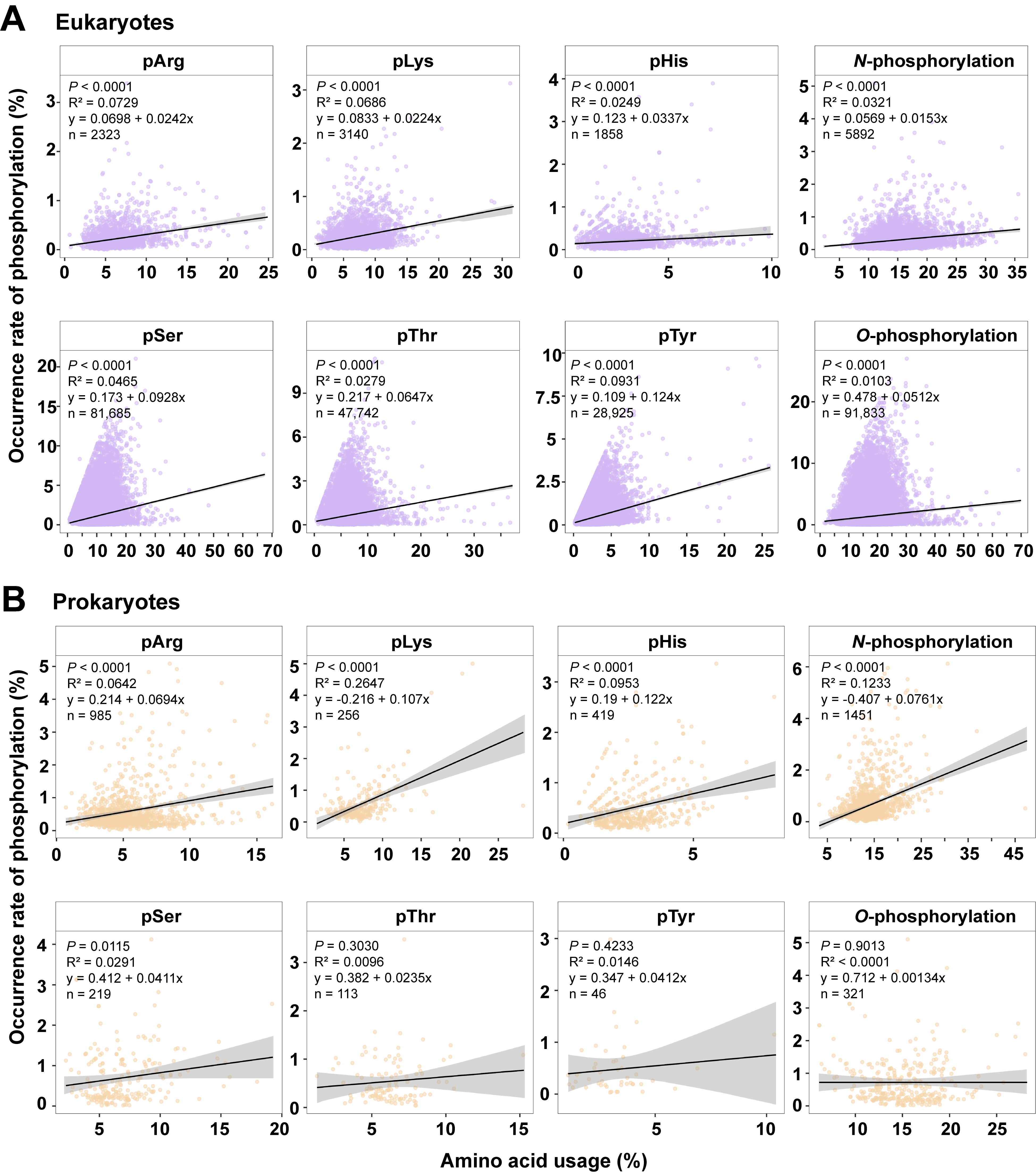

Supplement: qzae032_Supplementary_Data [file qzae032_supplementary_data.zip › Figure S2_final version20240328.tif]

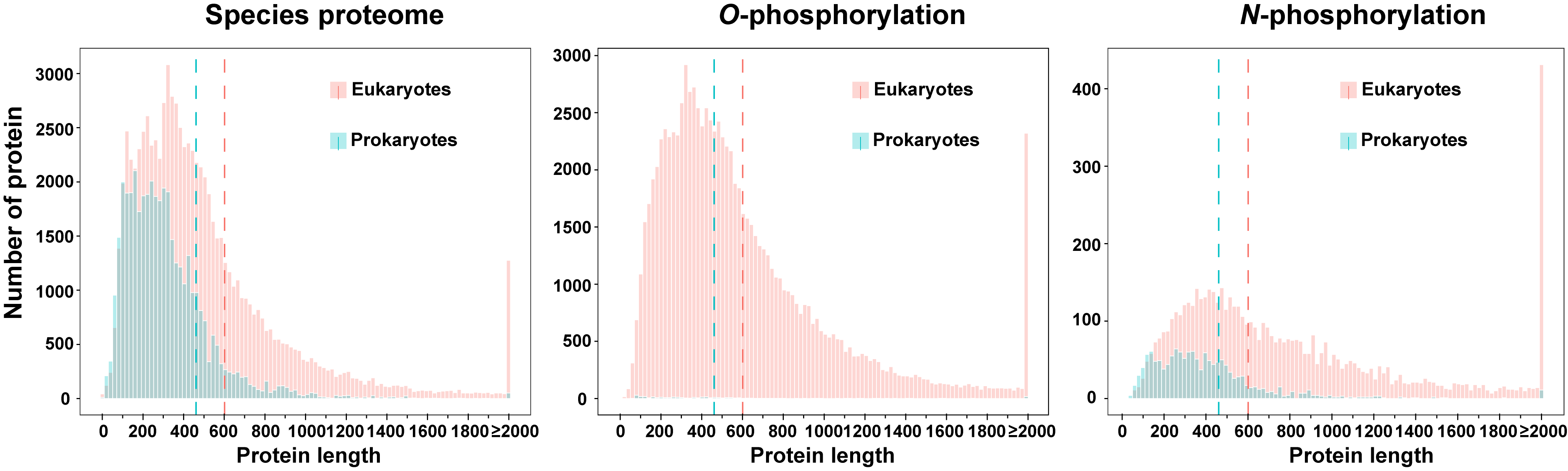

Supplement: qzae032_Supplementary_Data [file qzae032_supplementary_data.zip › Figure S1_final version20240328.tif]

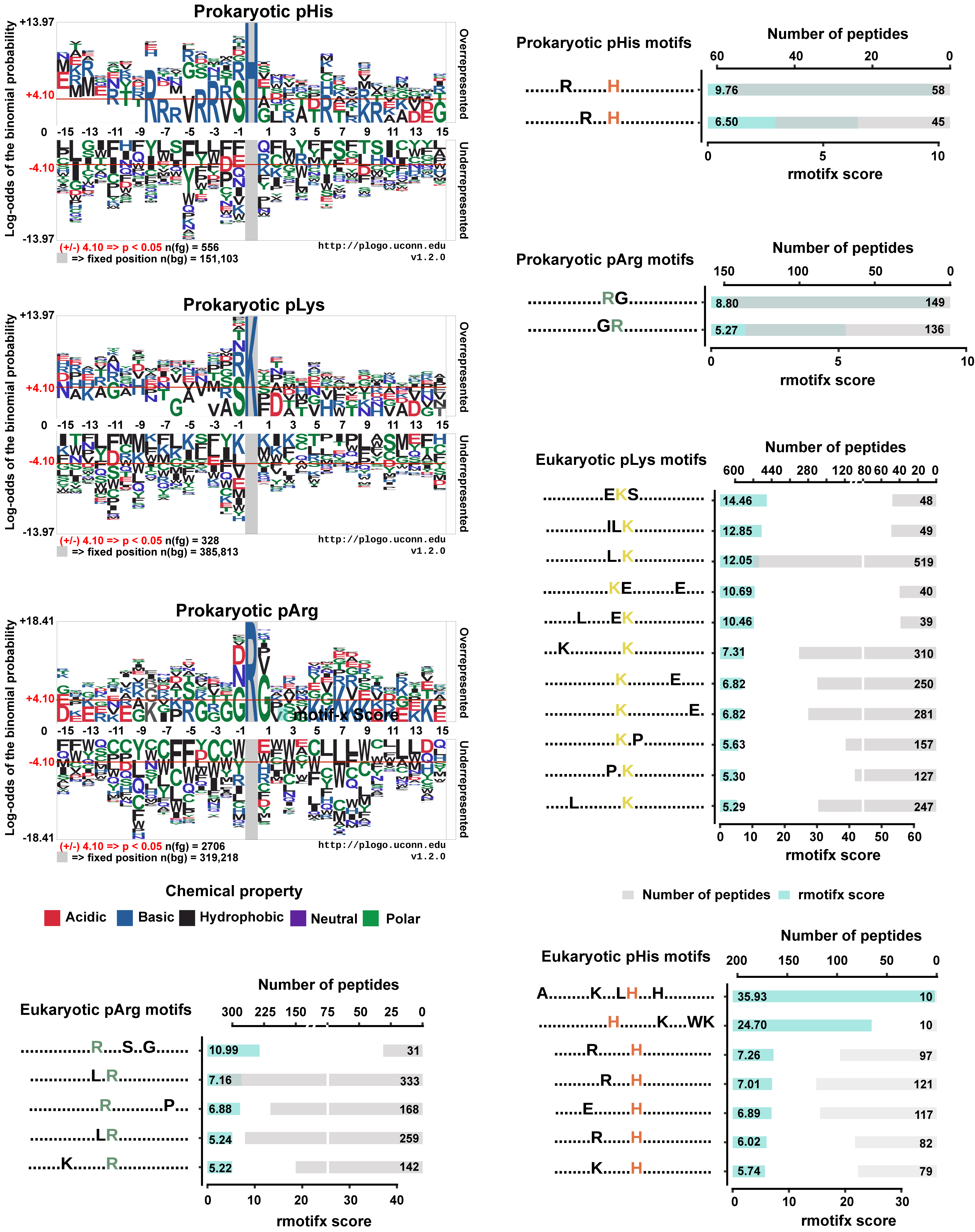

Supplement: qzae032_Supplementary_Data [file qzae032_supplementary_data.zip › Figure S3_final version20240328.tif]
